# Supplementary material for: Evidence supported by Mendelian randomization: impact on inflammatory factors in knee osteoarthritis
Source: Front Med (Lausanne). 2024 May 28;11:1382836. doi: 10.3389/fmed.2024.1382836 (PMC11165061; doi:10.3389/fmed.2024.1382836)
Supplement: Supplementary file 1 [file Data_Sheet_1.ZIP › data/GCST90274786_5e6/786.forest.pdf]

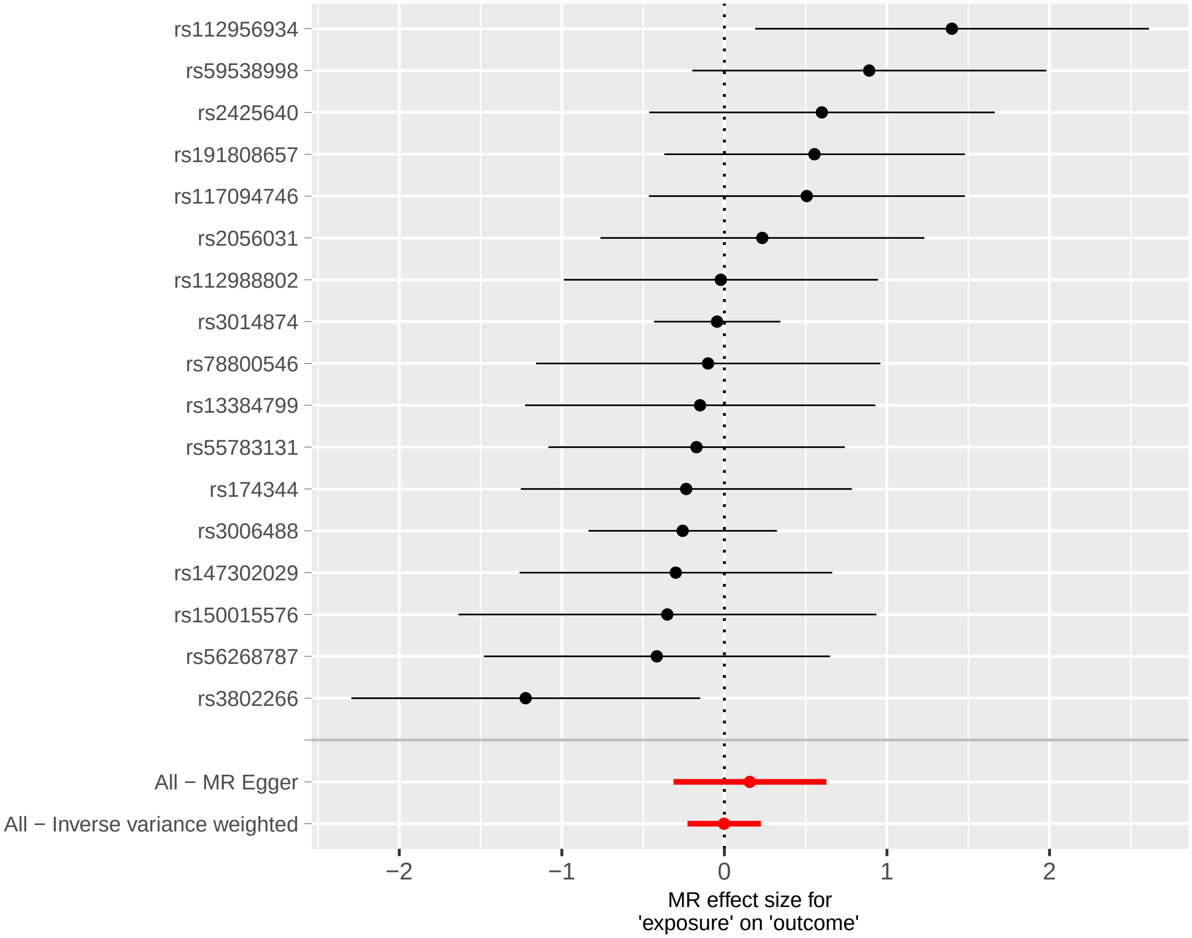

rs112956934
rs59538998
rs2425640
rs191808657
rs117094746
rs2056031
rs112988802
rs3014874
rs78800546
rs13384799
rs55783131
rs174344
rs3006488
rs147302029
rs150015576
rs56268787
rs3802266
All − MR Egger
All − Inverse variance weighted
−2
−1
0
1
2
MR effect size for
'exposure' on 'outcome'
